# Supplementary material for: The Phenotype of the Adipocytes Derived from Subcutaneous and Visceral ADMSCs Is Altered When They Originate from Morbidly Obese Women: Is There a Memory Effect?
Source: Cells. 2022 Apr 23;11(9):1435. doi: 10.3390/cells11091435 (PMC9099624; doi:10.3390/cells11091435)
Supplement: Supplementary file 1 [file cells-11-01435-s001.zip › cells-1675374-supplementary.pdf]

Supplementary Materials:

Figure S1. Gating strategy for the determination of the size and granularity of ADMSC-derived adipocytes by flow cytometry. Numbers indicate ranges used to classify the cells to different groups based on the cell size (I: 0-275, II: 275-550, III: 550-825, IV: 825-1100) or granularity (I: 0-250, II: 250-500, III: 500-750, IV: 750-1100).

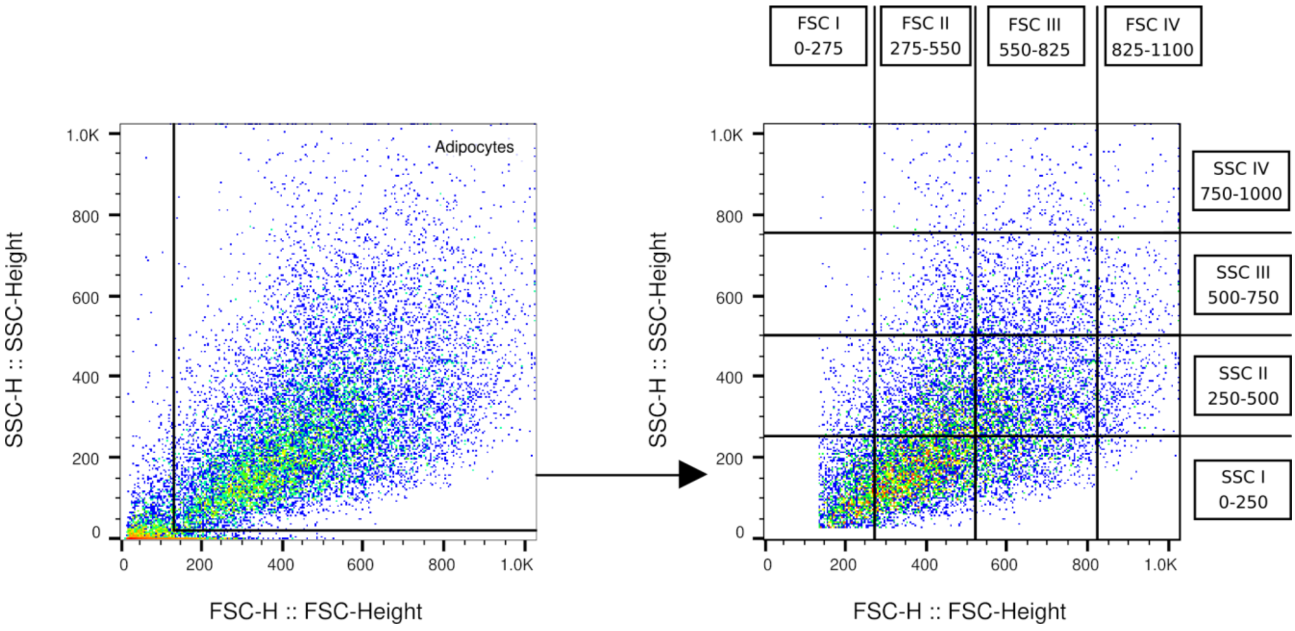

Table S1. The primers used for real-time PCR.

**Target gene**	**Forward primer (5'-3')**	**Reverse primer (5'-3')**	**Amplicon product size (bp)**
**TBC1D1**	GTGTGGGAAAAGATGCTTAGCA	GTGATGACGTGGCACACCTT	99
**TBC1D4**	AGCTCCAGTGAACAGTGCAGTG	CACTTAGGGACTCATTGCTGC	76
**CD36/SR-B2**	GGTACAGATGCAGCCTCATT	AGGCCTTGGATGGAAGAACA	157
**FATP1**	GCTAAGGCCCTGATCTTTGG	CCAAGTCTCCAGAGCAGAAC	100
**FATP4**	TGGCGCTTCATCCGGGTCTT	CGAACGGTAGAGGCAAACAA	140
**FABPpm**	GAAGGCAAAGGTGCGACAGT	GCCGAACGGTAGAGGCAAA	71
**RPLO13A**	CTATGACCAATAGGAAGAGCAACC	GCAGAGTATATGACCAGGTGGAA	121

Table S2. Clinical characteristics of patients (tissue donors).

	**Lean**	**Obese(-)**	**Obese(+)**
**Age [years]**	58.0 ± 3.367	58.25 ± 2.63	59.0 ± 3.162
**BMI [kg/m^2]**	24.85 ± 3.104	42.38 ± 2.416 a	50.19 ± 4.509 ab
**Waist circumference [cm]**	77.5 ± 7.0	117.5 ± 4.041 a	139.5 ± 17.02 a
**WHR**	0.84 ± 0.138	0.92 ± 0.028	0.93 ± 0.026
**CRP [mg/L]**	5.48 ± 0.435	9.19 ± 0.848 a	12.28 ± 2.457 a
**Glucose [mg/dL]**	72.75 ± 2.217	99.75 ± 12.894 a	104.5 ± 11.733 a
**Insulin [uIU/mL]**	7.62 ± 0.672	20.52 ± 2.622 a	28.14 ± 7.148 a
**HOMA-IR**	1.37 ± 0.085	5.04 ± 0.812 a	7.35 ± 2.415 a
**ALT [IU/L]**	24.5 ± 4.041	36.75 ± 6.344 a	29.25 ± 1.893
**AST [IU/L]**	23.0 ± 2.0	20.5 ± 3.697	34.25 ± 8.221 b
**Chol [mg/dL]**	170.75 ± 6.702	171.25 ± 11.529	207.25 ± 57.996
**LDL [mg/dL]**	114.75 ± 1.5	123.5 ± 9.147	140.25 ± 35.929
**TAG [mg/dL]**	138.5 ± 4.655	145.5 ± 6.455	171.0 ± 19.201 a
**HDL [mg/dL]**	60.25 ± 6.238	50.25 ± 4.272 a	38.0 ± 12.41 a
**WBC [10^3/μL]**	7.76 ± 0.668	8.4 ± 1.962	7.95 ± 1.666
**RBC [10^3/μL]**	4.81 ± 0.149	4.66 ± 0.405	4.95 ± 0.285
**HGB [g/dL]**	14.9 ± 0.849	14.1 ± 0.913	13.68 ± 0.793
**PLT [10^3/μL]**	287.5 ± 11.902	289.25 ± 50.129	308.75 ± 23.128
**Fibrynogen [mg/dL]**	379.5 ± 9.0	404.0 ± 19.9	429.75 ± 9.106 a
**SP [mmHg]**	126.25 ± 9.465	128.75 ± 11.087	143.75 ± 9.465 a
**DP [mmHg]**	80.0 ± 4.082	86.25 ± 4.787	95.0 ± 4.082 ab

Data are presented as mean ± SD.

$p < 0.05$; a – indicate significant differences from lean subjects; b – indicate significant differences from Obese(-) group.

ALT – alanine transaminase; AST – asparatate transaminase; BMI – body mass index; Chol – cholesterol; CRP – C-reactive protein; DP – diastolic pressure; HDL – high density lipoprotein; HGB – hemoglobin; HOMA-IR – homeostatic model assessment of insulin resistance; INR – international normalized ratio; LDL – low-density

lipoprotein; PLT – platelet count; RBC – red blood cell count; SP – systolic pressure; TAG – triacylgycerol; WBC – white blood cell count; WHR – waist-hip ratio.

Table S3. FFA—Fatty acid composition (nmol/mg of protein).

	subLean	subObese(-)	subObese(+)	visLean	visObese(-)	visObese(+)
**Myristic acid (14:0)**	1.85 ± 0.868	3.7 ± 0.546 a	4.4 ± 2.157	2.37 ± 0.623	2.32 ± 0.536 c	2.37 ± 0.966
**Palmitic acid (16:0)**	17.49 ± 2.708	18.27 ± 4.952	26.6 ± 4.661 a	15.43 ± 4.469	16.92 ± 8.007	12.94 ± 4.687 c
**Palmitooleic acid (16:1)**	5.94 ± 0.468	6.59 ± 0.856	11.17 ± 5.651	4.6 ± 1.565	4.81 ± 1.46	1.65 ± 0.956 abc
**Stearic acid (18:0)**	7.14 ± 1.647	5.48 ± 1.903	6.5 ± 1.399	5.58 ± 2.109	6.91 ± 4.49	4.43 ± 1.232
**Oleic acid (18:1n9c)**	3.12 ± 0.738	2.76 ± 0.801	4.19 ± 2.059	1.88 ± 0.315 c	1.74 ± 0.365	1.23 ± 0.564
**Linoleic acid (18:2n6c)**	0.2 ± 0.183	0.14 ± 0.085	0.14 ± 0.015	0.13 ± 0.03	0.22 ± 0.065	0.15 ± 0.021
**Arachidic acid (20:0)**	0.16 ± 0.114	0.13 ± 0.055	0.12 ± 0.006	0.12 ± 0.023	0.15 ± 0.05	0.09 ± 0.018
**Linolenic acid (C18:9n3)**	0.18 ± 0.119	0.16 ± 0.08	0.11 ± 0.022	0.13 ± 0.024	0.19 ± 0.099	0.12 ± 0.026
**Behenic acid (22:0)**	0.12 ± 0.13	0.1 ± 0.053	0.07 ± 0.03	0.1 ± 0.017	0.11 ± 0.079	0.05 ± 0.008 a
**Arachidonic acid (20:4n6)**	0.23 ± 0.11	0.13 ± 0.078	0.17 ± 0.018	0.26 ± 0.039	0.19 ± 0.116	0.12 ± 0.027 ac
**Lignoceric acid (24:0)**	0.42 ± 0.26	0.15 ± 0.039 a	0.22 ± 0.111	0.44 ± 0.194	0.3 ± 0.141	0.19 ± 0.095
**Eicosapentaenoic acid (20:5n3)**	0.14 ± 0.067	0.47 ± 0.765	0.16 ± 0.075	0.07 ± 0.017	0.2 ± 0.127	0.12 ± 0.064
**Nervonic acid (24:1)**	0.09 ± 0.087	0.06 ± 0.026	0.04 ± 0.009	0.05 ± 0.008	0.06 ± 0.044	0.04 ± 0.009
**Docosahexaenoic acid (22:6n3)**	0.11 ± 0.069	0.09 ± 0.042	0.07 ± 0.009	0.09 ± 0.012	0.14 ± 0.065	0.08 ± 0.024
**Saturated**	27.18 ± 3.945	27.83 ± 7.002	37.9 ± 3.591 a	24.04 ± 6.82	26.72 ± 12.369	20.07 ± 6.899 c
**Unsaturated**	10.01 ± 0.44	10.39 ± 2.18	16.06 ± 7.723	7.21 ± 1.761 c	7.55 ± 1.979	3.52 ± 1.49 abc
**MUFA**	9.15 ± 0.267	9.41 ± 1.321	15.4 ± 7.667	6.53 ± 1.715	6.61 ± 1.803	2.93 ± 1.515 abc
**PUFA**	0.86 ± 0.542	0.98 ± 0.946	0.66 ± 0.06	0.68 ± 0.074	0.93 ± 0.372	0.59 ± 0.027
**Total**	37.19 ± 4.323	38.22 ± 7.762	53.96 ± 10.07 a	31.26 ± 5.15	34.27 ± 10.864	23.58 ± 8.355 c

a – difference vs. Lean group in the studied tissue; b – difference vs. Obese(-) group in the studied tissue; c – difference between adipocytes differentiated from visADMSCs vs. subADMSCs within the patient donor metabolic status. Data are presented as mean ± SD (n=4 for each study group, measurements taken in triplicate). $P < 0.05$. Designation of the groups: Obese(-) – obese without metabolic syndrome patients; Obese(+) – obese with metabolic syndrome patients.

Table S4. DAG—Fatty acid composition (nmol/mg of protein).

	subLean	subObese(-)	subObese(+)	visLean	visObese(-)	visObese(+)
**Myristic acid (14:0)**	4.75 ± 0.614	4.88 ± 1.407	7.89 ± 1.78 ab	6.87 ± 1.916	6.22 ± 1.138	5.21 ± 2.028
**Palmitic acid (16:0)**	19.81 ± 3.129	23.17 ± 6.66	31.59 ± 7.534 a	23.68 ± 6.945	22.86 ± 5.498	19.74 ± 12.48
**Palmitooleic acid (16:1)**	10.98 ± 1.508	14.07 ± 1.69	17.87 ± 7.299	9.86 ± 7.645	9.57 ± 2.785 c	3.38 ± 2.29 c
**Stearic acid (18:0)**	4.11 ± 2.327	5.99 ± 3.413	10.99 ± 10.569	4.93 ± 2.104	7.16 ± 3.699	7.81 ± 4.914
**Oleic acid (18:1n9c)**	5.13 ± 0.812	6.55 ± 1.527	7.07 ± 2.314	5.24 ± 2.304	4.31 ± 0.468	2.79 ± 1.786 c
**Linoleic acid (18:2n6c)**	0.22 ± 0.102	0.31 ± 0.184	0.39 ± 0.067 a	0.42 ± 0.236	0.49 ± 0.092	0.39 ± 0.152
**Arachidic acid (20:0)**	0.19 ± 0.072	0.2 ± 0.099	0.22 ± 0.056	0.2 ± 0.065	0.28 ± 0.067	0.26 ± 0.118
**Linolenic acid (C18:9n3)**	0.16 ± 0.05	0.27 ± 0.118	0.24 ± 0.021	0.18 ± 0.103	0.27 ± 0.086	0.24 ± 0.083
**Behenic acid (22:0)**	0.12 ± 0.014	0.15 ± 0.073	0.16 ± 0.063	0.18 ± 0.071	0.16 ± 0.075	0.17 ± 0.082
**Arachidonic acid (20:4n6)**	0.7 ± 0.322	0.53 ± 0.31	0.61 ± 0.418	0.87 ± 0.31	0.58 ± 0.388	0.52 ± 0.141 a
**Lignoceric acid (24:0)**	0.28 ± 0.095	0.23 ± 0.052	0.23 ± 0.124	0.48 ± 0.189	0.37 ± 0.149	0.35 ± 0.161
**Eicosapentaenoic acid (20:5n3)**	0.11 ± 0.055	0.26 ± 0.279	0.22 ± 0.096	0.16 ± 0.019	0.3 ± 0.158	0.22 ± 0.077
**Nervonic acid (24:1)**	0.07 ± 0.038	0.08 ± 0.037	0.07 ± 0.015	0.1 ± 0.045	0.11 ± 0.074	0.12 ± 0.083
**Docosahexaenoic acid (22:6n3)**	0.08 ± 0.044	0.14 ± 0.078	0.14 ± 0.025	0.16 ± 0.026 c	0.25 ± 0.071	0.21 ± 0.074
**Saturated**	29.24 ± 5.621	34.61 ± 10.476	51.08 ± 17.214	36.35 ± 8.652	37.05 ± 7.98	33.55 ± 17.871
**Unsaturated**	17.47 ± 2.076	22.21 ± 3.236	26.61 ± 9.549	17.0 ± 9.834	15.88 ± 3.355 c	7.87 ± 4.04 bc
**MUFA**	16.19 ± 2.202	20.7 ± 2.979	25.01 ± 9.199	15.2 ± 9.885	14.0 ± 3.082 c	6.3 ± 4.028 b
**PUFA**	1.28 ± 0.16	1.51 ± 0.829	1.6 ± 0.564	1.8 ± 0.268 c	1.88 ± 0.544	1.58 ± 0.451
**Total**	46.71 ± 4.51	56.82 ± 12.02	77.68 ± 7.932 ab	53.34 ± 15.555	52.94 ± 5.007	41.42 ± 21.817 c

a – difference vs. Lean group in the studied tissue; b – difference vs. Obese(-) group in the studied tissue; c – difference between adipocytes differentiated from visADMSCs vs. subADMSCs within the patient donor metabolic status. Data are presented as mean ± SD (n=4 for each study group, measurements taken in triplicate). $P < 0.05$. Designation of the groups: Obese(-) – obese without metabolic syndrome patients; Obese(+) – obese with metabolic syndrome patients.

Table S5. TAG—Fatty acid composition (nmol/mg of protein).

	subLean	subObese(-)	subObese(+)	visLean	visObese(-)	visObese(+)
**Myristic acid (14:0)**	296.95 ± 118.385	279.89 ± 105.482	405.53 ± 35.818	141.82 ± 70.563	176.43 ± 54.541	105.96 ± 54.966 c
**Palmitic acid (16:0)**	1136.74 ± 280.446	905.26 ± 447.232	1597.02 ± 260.068 b	534.39 ± 304.508 c	769.51 ± 178.888	473.76 ± 144.096 bc
**Palmitooleic acid (16:1)**	1149.78 ± 278.541	893.83 ± 402.716	1518.93 ± 329.644	476.04 ± 330.707	645.3 ± 87.783	326.88 ± 163.308 bc
**Stearic acid (18:0)**	40.69 ± 23.224	45.91 ± 35.277	55.97 ± 28.863	21.42 ± 5.789	18.87 ± 6.33	23.95 ± 5.48
**Oleic acid (18:1n9c)**	752.18 ± 243.702	530.68 ± 249.722	788.92 ± 67.238	252.16 ± 116.216 c	286.23 ± 41.702	199.35 ± 86.299 c
**Linoleic acid (18:2n6c)**	4.95 ± 0.706	4.65 ± 2.373	6.42 ± 1.519	2.16 ± 0.377 c	3.94 ± 1.5	2.85 ± 1.661 c
**Arachidic acid (20:0)**	0.9 ± 0.192	0.98 ± 0.492	1.48 ± 0.341 a	0.66 ± 0.174	0.73 ± 0.218	0.79 ± 0.33 c
**Linolenic acid (C18:9n3)**	11.49 ± 1.193	9.96 ± 2.986	15.36 ± 3.632	5.44 ± 1.73 c	7.97 ± 1.366	6.17 ± 0.879 c
**Behenic acid (22:0)**	4.49 ± 7.471	4.09 ± 3.934	6.39 ± 3.357	1.63 ± 2.16	4.12 ± 2.519	3.72 ± 2.656
**Arachidonic acid (20:4n6)**	5.27 ± 1.522	4.95 ± 1.806	5.51 ± 1.494	3.62 ± 0.571	4.56 ± 0.703	3.16 ± 1.24
**Lignoceric acid (24:0)**	1.18 ± 0.79	1.12 ± 1.114	0.72 ± 0.4	0.72 ± 0.406	1.07 ± 1.014	1.16 ± 1.558
**Eicosapentaenoic acid (20:5n3)**	3.47 ± 1.253	1.08 ± 1.188	1.94 ± 2.048	2.22 ± 1.117	0.59 ± 0.191	1.83 ± 1.683
**Nervonic acid (24:1)**	0.72 ± 0.232	0.65 ± 0.362	0.76 ± 0.257	0.33 ± 0.028 c	0.65 ± 0.212 a	0.87 ± 0.633 a
**Docosahexaenoic acid (22:6n3)**	3.92 ± 1.234	5.28 ± 2.142	4.83 ± 2.352	4.1 ± 1.536	6.04 ± 2.778	6.53 ± 3.231
**Saturated**	1480.95 ± 369.956	1237.25 ± 581.334	2067.11 ± 282.823 a	700.63 ± 364.874 c	970.72 ± 230.722	609.34 ± 196.875 c
**Unsaturated**	1931.76 ± 239.881	1451.08 ± 632.778	2342.67 ± 348.505	746.07 ± 445.475 c	955.29 ± 130.694	547.63 ± 252.659 bc
**MUFA**	1902.67 ± 238.386	1425.16 ± 628.189	2308.6 ± 346.469	728.52 ± 446.652 c	932.18 ± 129.041	527.1 ± 247.998 c
**PUFA**	28.37 ± 3.588	25.85 ± 7.417	34.43 ± 4.78	17.82 ± 2.035 c	23.2 ± 4.307	20.3 ± 5.097 c
**Total**	3412.72 ± 465.64	2688.33 ± 1194.037	4409.78 ± 598.482 a	1446.7 ± 806.721 c	1926.01 ± 325.215	1156.97 ± 446.993 bc

a – difference vs. Lean group in the studied tissue; b – difference vs. Obese(-) group in the studied tissue; c – difference between adipocytes differentiated from visADMSCs vs. subADMSCs within the patient donor metabolic status. Data are presented as mean ± SD (n=4 for each study group, measurements taken in triplicate).

P < 0.05. Designation of the groups: Obese(-) – obese without metabolic syndrome patients; Obese(+) – obese with metabolic syndrome patients.

Table S6. Summary table of ANOVA analysis.

Three-way ANOVA		
TBC1D1 mRNA		
Source of variation	% of total variance	p value
tissue	0.8	0.7
obesity	0.1	0.9
metabolic syndrome	1.2	0.63
residual	98	
Three-way ANOVA		
TBC1D4/AS160 mRNA		
Source of variation	% of total variance	p value
tissue	25.2	< 0.01
obesity	35.2	< 0.01
metabolic syndrome	1.4	0.4
residual	38.2	
Three-way ANOVA		
TBC1D1 protein		
Source of variation	% of total variance	p value
tissue	82	< 0.01
obesity	0.2	0.83
metabolic syndrome	16.8	0.08
residual	0.9	
Three-way ANOVA		
TBC1D4/AS160 protein		
Source of variation	% of total variance	p value
tissue	43	< 0.01
obesity	29.6	< 0.01
metabolic syndrome	1.4	0.31
residual	26.1	
Three-way ANOVA		
palmitate uptake [dpm/mg of protein]		
Source of variation	% of total variance	p value
tissue	15.7	< 0.01
obesity	13.4	0.01

metabolic syndrome	37.6	< 0.01
residual	33.3	
Three-way ANOVA		
FAT/CD36 mRNA		
Source of variation	% of total variance	p value
tissue	4.8	0.18
obesity	0.5	0.65
metabolic syndrome	45.1	< 0.01
residual	49.6	
Three-way ANOVA		
FABPpm mRNA		
Source of variation	% of total variance	p value
tissue	31.7	< 0.01
obesity	1.8	0.47
metabolic syndrome	0.3	0.78
residual	66.2	
Three-way ANOVA		
FATP1 mRNA		
Source of variation	% of total variance	p value
tissue	9.7	0.15
obesity	1.6	0.55
metabolic syndrome	0.7	0.69
residual	88	
Three-way ANOVA		
FATP4 mRNA		
Source of variation	% of total variance	p value
tissue	21.3	0.02
obesity	8.1	0.13
metabolic syndrome	4.5	0.26
residual	66.1	
Three-way ANOVA		
FAT/CD36 protein		
Source of variation	% of total variance	p value
tissue	68.4	< 0.01
obesity	9.4	< 0.01
metabolic syndrome	6.4	0.01
residual	15.8	

Three-way ANOVA		
FABPpm protein		
Source of variation	% of total variance	p value
tissue	42.1	0.2
obesity	12.6	0.48
metabolic syndrome	8	0.568
residual	37.3	
Three-way ANOVA		
FATP1 protein		
Source of variation	% of total variance	p value
tissue	1.3	0.48
obesity	44.1	< 0.01
metabolic syndrome	1.5	0.46
residual	53	
Three-way ANOVA		
FATP4 protein		
Source of variation	% of total variance	p value
tissue	1.4	0.54
obesity	20.4	0.03
metabolic syndrome	4.9	0.26
residual	73.3	
Three-way ANOVA		
FFA (total)		
Source of variation	% of total variance	p value
tissue	33.6	< 0.01
obesity	1.8	0.46
metabolic syndrome	0.8	0.62
residual	63.8	
Three-way ANOVA		
DAG (total)		
Source of variation	% of total variance	p value
tissue	12.4	0.1
obesity	4.6	0.3
metabolic syndrome	1.4	0.56
residual	81.5	
Three-way ANOVA		
TAG (total)		

Source of variation	% of total variance	p value
tissue	59.8	< 0.01
obesity	0.2	0.76
metabolic syndrome	2.3	0.29
residual	37.8	
Four-way ANOVA		
ROS [mg/mL]		
Source of variation	% of total variance	p value
tissue	3.9	< 0.01
obesity	2.7	0.03
metabolic syndrome	0.1	0.62
differentiation	71.5	< 0.01
residual	21.8	
Three-way ANOVA		
FASN protein		
Source of variation	% of total variance	p value
tissue	55.4	< 0.01
obesity	23.2	< 0.01
metabolic syndrome	0	0.86
residual	21.4	
Three-way ANOVA		
DGAT1 protein		
Source of variation	% of total variance	p value
tissue	64.9	< 0.01
obesity	20.9	< 0.01
metabolic syndrome	2	0.09
residual	12.2	
Three-way ANOVA		
β-HAD protein		
Source of variation	% of total variance	p value
tissue	29.2	< 0.01
obesity	25.6	< 0.01
metabolic syndrome	15.4	< 0.01
residual	29.8	
Three-way ANOVA		
ATGL		
Source of variation	% of total variance	p value

tissue	62.6	< 0.01
obesity	0.1	0.77
metabolic syndrome	2.7	0.23
residual	34.6	
